# The prevalence of SARS-CoV-2 antibodies within the community of a private tertiary university in the Philippines: A serial cross sectional study

**DOI:** 10.1371/journal.pone.0268145

**Published:** 2022-12-05

**Authors:** Lourdes Bernadette C. Sumpaico-Tanchanco, Jenica Clarisse Y. Sy, Angel Belle C. Dy, Myla Levantino, Arianna Maever L. Amit, John Wong, Kirsten Angeles, John Paul C. Vergara

**Affiliations:** 1 School of Medicine and Public Health, Ateneo de Manila University, Quezon City, Philippines; 2 National Clinical Trials and Translation Center, National Institutes of Health, University of the Philippines Manila, Manila, Philippines; 3 EpiMetrics, Inc, Manila, Philippines; 4 Professional Schools, Ateneo de Manila University, Manila, Philippines; University of the Witwatersrand, SOUTH AFRICA

## Abstract

The COVID-19 pandemic has caused a public health emergency in all sectors of society, including universities and other academic institutions. This study determined the seroprevalence of SARS-CoV-2 antibodies among administrators, faculty, staff, and students of a private tertiary academic institution in the Philippines over a 7 month period. It employed a serial cross-sectional method using qualitative and quantitative COVID-19 antibody test kits. A total of 1,318 participants were tested, showing 47.80% of the study population yielding IgG antibodies to SARS-CoV-2 virus. A general increase in seroprevalence was observed from June to December 2021, which coincided with the vaccine roll-out of the country. All brands yielded positive antibody formation, with mRNA vaccines having higher levels than other types of vaccines. A decreasing trend in IgG reactivity was found in vaccinated individuals after 1 to 6 months of completion of the 2 doses of the COVID-19 vaccine. Where possible, IgG and T-cell reactivity and/or neutralizing capacity against SAR-CoV-2 need to be monitored regardless of vaccine brand. Together with uptake of COVID-19 vaccines and boosters, other public health interventions such as wearing of masks and regular testing need to be continued for better protection. Effective communication is also needed to inform risks associated with activities across different settings. Investments in long-term measures such as air filtration and ventilation systems, and wastewater surveillance need to be made.

## Introduction

Coronavirus disease (COVID-19) is caused by the severe acute respiratory syndrome coronavirus 2 (SARS-CoV-2) virus which has infected millions of people. A comprehensive understanding of the epidemiology of the virus, accurate measurement and reporting of the extent of transmission and infection are important to inform government response [1, 2].

Serology, or antibody testing determines the presence of antibodies produced against SARS-CoV-2 in an individual’s blood sample [3]. It is a reliable diagnostic alternative that can be used where other tests are not available [4]. It involves a simple lateral flow test that only requires a small amount of blood. It can be used to detect binding antibodies and identify recovered cases through remaining IgG antibodies [5]. SARS-CoV-2 infected patients mostly exhibit an antibody response 10 to 15 days after infection, and there is a sequential or simultaneous seroconversion for immunoglobulin G (IgG) and immunoglobulin M (IgM) [6]. Quantitative antibody tests may determine antibody titers, enable longitudinal monitoring of antibody levels in patients, and potentially monitor antibody response to vaccines.

Seroprevalence surveys estimate the percentage of individuals in a population who have antibodies against the virus at a large scale. Serological testing is ideal in approximating cumulative prevalence because antibodies against SARS-CoV-2 persist for a longer amount of time, after infection [7]. Compared to the viral load detected by the real-time reverse transcription polymerase chain reaction (RT-PCR) testing, antibodies, more specifically IgG antibodies, persist for an extended period of time even after the infection has been cleared. More specifically, IgG antibodies have been found to persist more than 3 months after infection [8].

Given its utility for pandemic planning and response, population-based seroprevalence studies have been conducted among different population groups, using different study designs and tests, in both hospital and community settings [1]. Seroprevalence studies have been conducted in hospital settings and community settings in countries across the world, such as China [7], Switzerland [9], Iran [10], Hong Kong [11], US [12], and Brazil [13], among others. Studies have been conducted cross sectionally, across different households and age groups, using immunofluorescence assays for anti-SARS-CoV-2 IgG and IgM antibodies. However, no published estimates are available in the Philippines since 2020, in part because of resource limitations. Antibody testing is instrumental in generating epidemiological information necessary for the control of infectious diseases, including COVID-19. The surveillance of COVID-19 is necessary in order to determine disease burden in the population and to detect vulnerable groups who are at high risk. Determining the prevalence of COVID-19 is important in understanding the widespread outbreak of the disease, transmission of the virus, and the immunity status of the population. In order to implement proper strategies to eradicate COVID-19, it is crucial to identify the number of people previously and currently infected with the virus, and who are most at risk.

The primary objective of this study was to determine the seroprevalence of antibody titers in employees in a tertiary academic university across a 6 month period. The secondary objective was to determine the association between sociodemographic characteristics, medical history, vaccine brand, COVID-19 testing positivity, and serological status. Finally, the study aimed to correlate qualitative and quantitative antibody testing.

## Materials and methods

### Study design

A serial cross-sectional study was conducted during the early implementation of the vaccination campaign from June to December 2021. Participants were recruited for a monthly qualitative COVID-19 rapid antibody test, except August and November when a more stringent lockdown was implemented that prohibited blood collection in the study site. In September, a quantitative COVID-19 antibody test was performed together with the qualitative COVID-19 rapid antibody test.

### Participant recruitment

Faculty, administrators, professionals, staff, maintenance, security guards, affiliates, in-campus residents, and students of a private tertiary university were invited to participate through official school channels. A sample size requirement of 500 with a detectable difference of 0.10, a level of significance of 0.05, and a 25% non-response rate was computed [14, 15].

### Data collection and monitoring instrument

BluEHR (Tantum Quantum Headquarters, Inc), a cloud-based electronic medical record (EMR), was used during data collection. All participants accomplished a study questionnaire consisting of questions related to each of their demographics, medical history, COVID-19 vaccination status, and COVID-19 precautionary behaviors.

### Sample collection and antibody testing

A medical professional (medical technologist or physician) collected blood samples from the participants. The SARS-CoV-2 Rapid Antibody Kit by Roche Diagnostics was used for the qualitative antibody testing. It has a specificity of 98.6% and sensitivity of 99.0% [16]. Twenty (20) μl of blood was obtained through finger pricking. The collected blood was added to the lateral flow test device, followed by 90 μl (approximately 3 drops) of buffer. The test was incubated for 10–15 mins before interpretation. Both IgG and IgM antibodies were detected by the rapid chromatographic immunoassay. Participants with reactive IgM results were referred to the university physician for confirmatory real-time reverse transcription polymerase chain reaction (RT-PCR) testing and monitoring.

The Elecsys Anti-SARS-COV-2 S Antibody testing kit by Roche Diagnostics was used for quantitative antibody testing in the 4^th^ month of collection. This assay quantitatively determined the presence of antibodies to the SARS-CoV-2 spike protein receptor binding domain. Three to 4 ml of blood were obtained from each participant through venipuncture. The samples were stored in a vacutainer tube and sent to an outsourced laboratory for testing while stored at 2–4°C. Results were interpreted as follows: values <0.8 U/mL are considered non-reactive, and values ≥0.8 U/mL are considered reactive. The outsourced laboratory is licensed for operation by the Department of Health (DOH) and is also registered at the National Privacy Commission.

### Data analysis

Seroprevalence was defined as the proportion of participants with a reactive IgG result in the qualitative rapid antibody test [17]. The data was analyzed using SPSS software (version 28.0.1.0) and Google Sheets.

Descriptive statistics were computed: percentage for categorical variables, and mean or median continuous variables. Association between variables was assessed using Chi-square test, independent t-tests, and one-way analysis of variance (ANOVA). Sociodemographic variables were categorized as follows: age (59 and younger, 60 and above), region of residence (National Capital Region or NCR and outside of NCR), highest educational attainment (college and postgraduate, and primary, secondary or others), and work arrangement (remote or on-site). Testing positive for COVID-19 and having a member of the household testing positive for COVID-19 were categorized into ‘yes’ and ‘no’. One-way ANOVA was used to analyze a relationship between the quantitative count and vaccine brands. Tukey’s HSD was used as a post hoc test.

To determine association between sociodemographic characteristics, medical history, vaccine brand, COVID-19 testing positivity, and serological status, logistic regression analysis was conducted. Results were considered statistically significant if p-values were less than 0.05.

In order to compare the results of the qualitative and quantitative antibody tests, a receiver operating characteristic (ROC) analysis was performed [18, 19]. The most plausible U/ml cutoff for the qualitative test kit was estimated by considering each measurement as a candidate cutoff point and computing the resulting accuracies. In this specific analysis, accuracy is defined as the percentage of correctly classified data points over N = 453, where “correctly classified” is determined by using the results obtained from the qualitative test as ground truth.

The analyses excluded participants who have had COVID-19 (n = 38) to determine the seropositivity rates caused by vaccination. COVID-19 infection was determined based on the self-report of the participants in the health questionnaire. Participants who received their vaccine more than 6 months prior to testing were also excluded due to the small sample size (n = 17). For association analysis of vaccine brand and IgG reactivity, Johnson and Johnson and Sputnik were excluded due to the small number of individuals who received them.

### Ethics statement

The study was reviewed and approved by the Institutional Review Board (IRB) from the School of Medicine and Public Health Panel of the Ateneo de Manila University (AdMU) Research Ethics Committee. Informed consent was secured prior to inclusion to the study through the digital data collection tool used in the study.

## Results

### Participant characteristics

A total of 1,318 participants were recruited to participate in the study. Almost an equal proportion of males and females participated in this study. The study population ages ranged from 20 to 87, with a median age of 38 years (IQR: 26–49). Majority of them were college graduates or with postgraduate degrees (87.5%). Work arrangements of the participants also varied in that 33.1% worked remotely, while 55.5% had onsite work. Almost half (49.5%) of participants reported having at least one comorbidity, with hypertension being the most common (Table 1).

**Table 1:** Sociodemographic characteristics and medical history of the study population (N = 1,318).

Variable	N (%)
Sex	
Female	648 (49.2)
Male	670 (50.8)
Age group	
18–59	1244 (94.4)
≥60	74 (5.6)
Role in the university	
Administration/Professional	179 (13.6)
Faculty	347 (26.3)
In-campus Residents	42 (3.2)
Maintenance/ Guards/ Subconcessional/ Outsourced/ Affiliates	211 (16)
Staff	267 (20.3)
Student	272 (20.6)
Educational attainment	
College/Postgraduate	1,153 (87.5)
Primary/Secondary education/Others	146 (11.1)
Missing	19 (1.4)
Work arrangement	
Remote	436 (33.1)
On-site	731 (55.5)
Missing	151 (11.4)
Area of residence	
National Capital Region (NCR)	1,074 (81.5)
Outside NCR	244 (18.5)
Comorbidities	
None	516 (39.2)
With at least 1 comorbidity	652 (49.5)
Diabetes	104 (7.9)
Hypertension	260 (19.7)
Obesity	137 (10.4)
Chronic obstructive pulmonary diseases (COPD)	1 (0.1)
Cardiovascular disease (CVD)	4 (0.3)
Asthma	84 (6.4)
Missing	150 (11.4)
Smoking status	
Smoker	97 (7.4)
Non-smoker	1,071 (81.3)
Missing	150 (11.3)

### COVID-19 antibody seroprevalence

**Fig 1:**
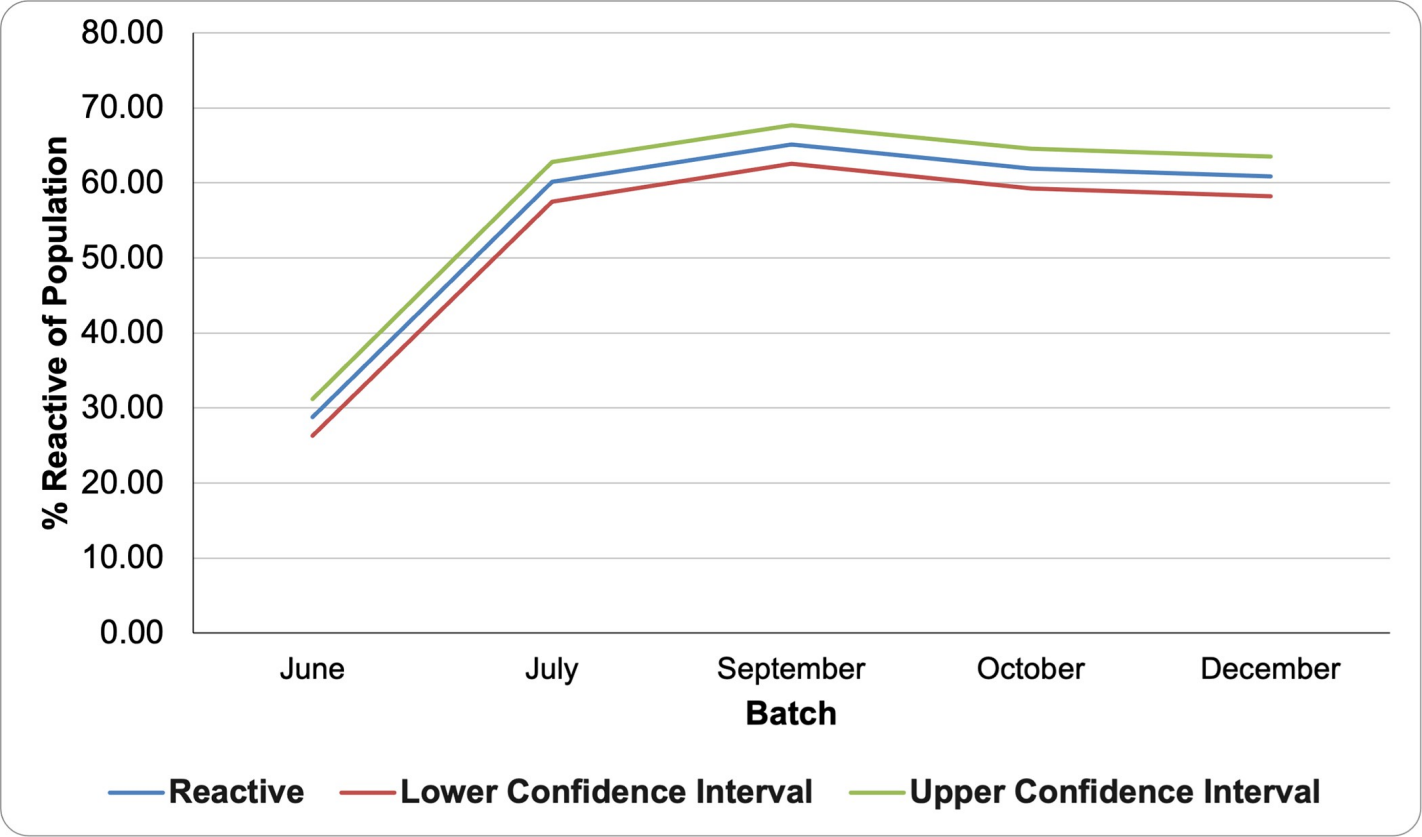
Seroprevalence of the study population across batches.

During the antibody testing, only 37 (2.8%) had reactive IgM serology results. Six hundred thirty (47.8%) of participants had reactive IgG serology results. Of those who received a reactive IgG result, 486 (77.1%) never tested positive for COVID-19 through RT-PCR. In this study population, only 38 (2.9%) participants previously tested positive for COVID-19 through RT-PCR, and 1,243 (94.3%) never tested positive. The seroprevalence of the study population ranged from 28.8% to 65.1%. A general increase in trend can be seen in the seroprevalence of participants over time (Fig 1).

**Fig 2:**
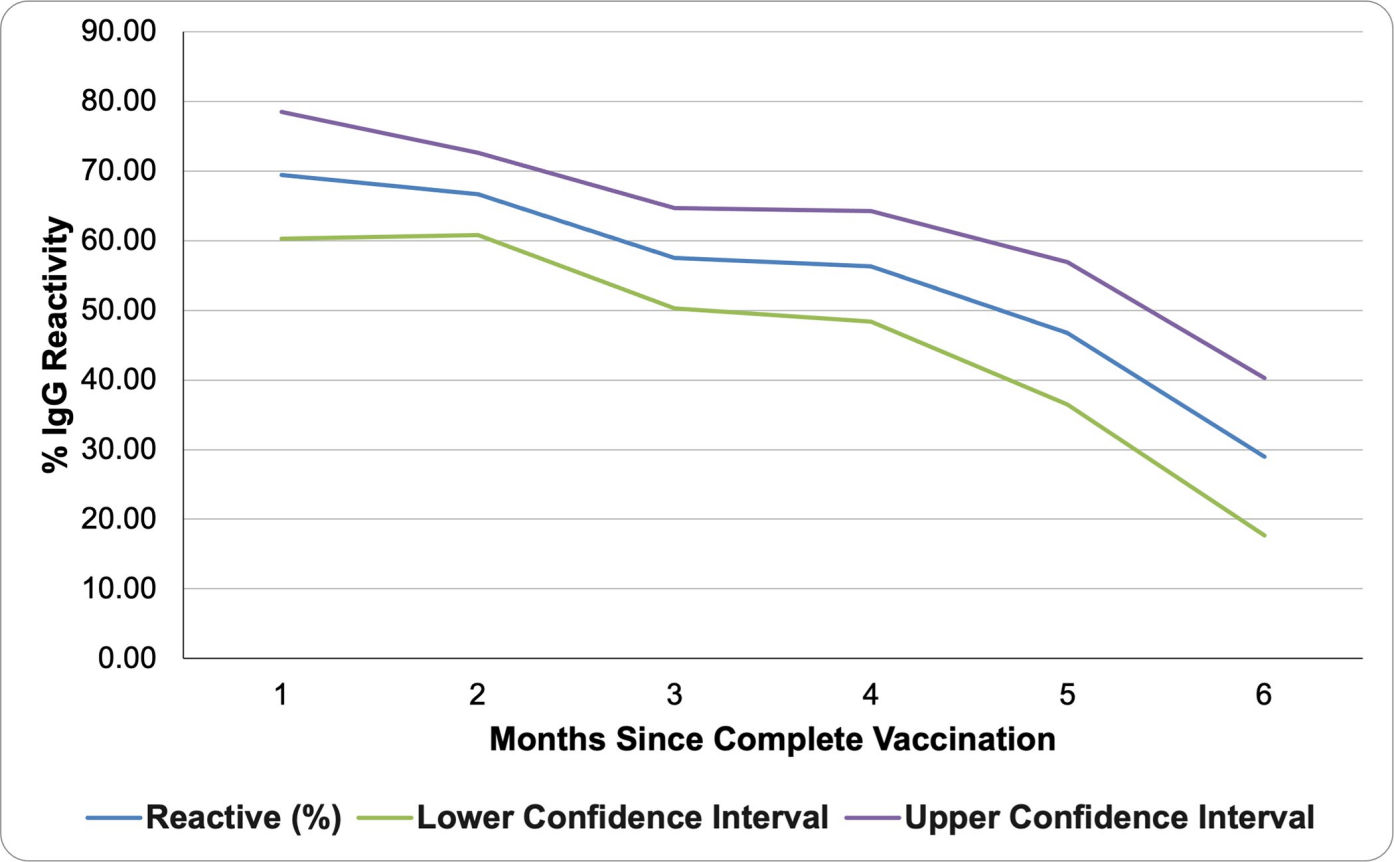
Percent of vaccinated respondents with reactive IgG results across time.

Participants who had COVID-19 (n = 38) were excluded from the analysis to determine the seropositivity rates caused by vaccination. COVID-19 infection was determined based on the self-report of the participants in the health questionnaire. Participants who received their vaccine more than 6 months prior to testing were also excluded due to the small sample size (n = 17). The seroprevalence of vaccinated members of the university community during the study period ranged from 28.8%-65.1%, across 6 months, shown in Fig 2. A general decrease in antibody levels was found over time measured from the last vaccination date. There was no significant difference in the seroprevalence of COVID-19 antibodies between males and females. Among men, the seroprevalence was 18.9%, and among women, it was 18.7%.

The total antibody titers ranged from 0.4 U/mL to >25,000 U/mL in the quantitative test (n = 409), excluding participants who were previously infected. The mean was 3,099.56 U/mL across all brands, with Moderna vaccine having the highest followed by Pfizer (Table 2).

**Table 2:** Descriptive summary of the quantitative antibody test results according to vaccine brand (N = 409).

Vaccine Brand	N	Mean (U/ml)	Std. Deviation	95% CI for Mean	
				Lower Bound	Upper Bound
Astrazeneca	88	2652.07	5308.56	1527.29	3776.85
Johnson and Johnson	4	2922.32	4439.41	-4141.76	9986.41
Moderna	29	7916.83	8177.15	4806.40	11027.25
Pfizer Biontech	28	3292.21	5314.19	1231.58	5352.84
CoronaVac	256	1591.17	4837.28	995.78	2186.55
Sputnik	4	931.37	450.38	214.72	1648.03
	409	2390.97	5468.42	1859.42	2922.51

### Correlation between qualitative and quantitative tests

**Fig 3:**
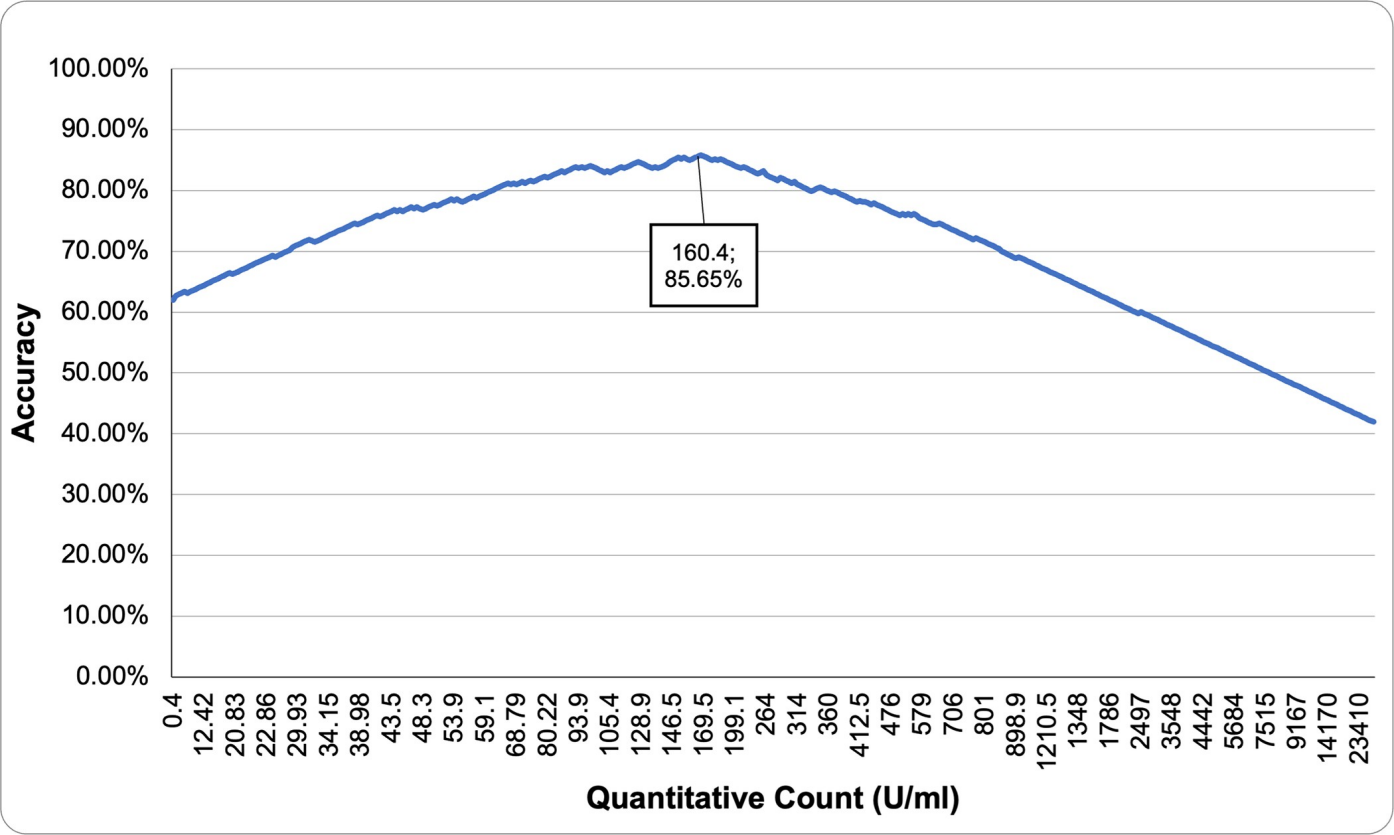
Percentage of accuracy for the quantitative antibody test cut off value.

The results of the qualitative antibody test were correlated with the quantitative test in order to identify a cutoff value at which participants received a reactive IgG result. Fig 3 displays the resulting accuracies against the candidate cutoff points and it reveals that a threshold of 160.4 U/ml yielded maximum accuracy (85.93%). This means that a positive result from the qualitative test occurs when at least 160.4 U/ml is detected from the specimen (note that the candidate value before this cutoff is 157.1 U/ml, so alternatively, the condition can be stated as > 157 U/ml).

**Fig 4:**
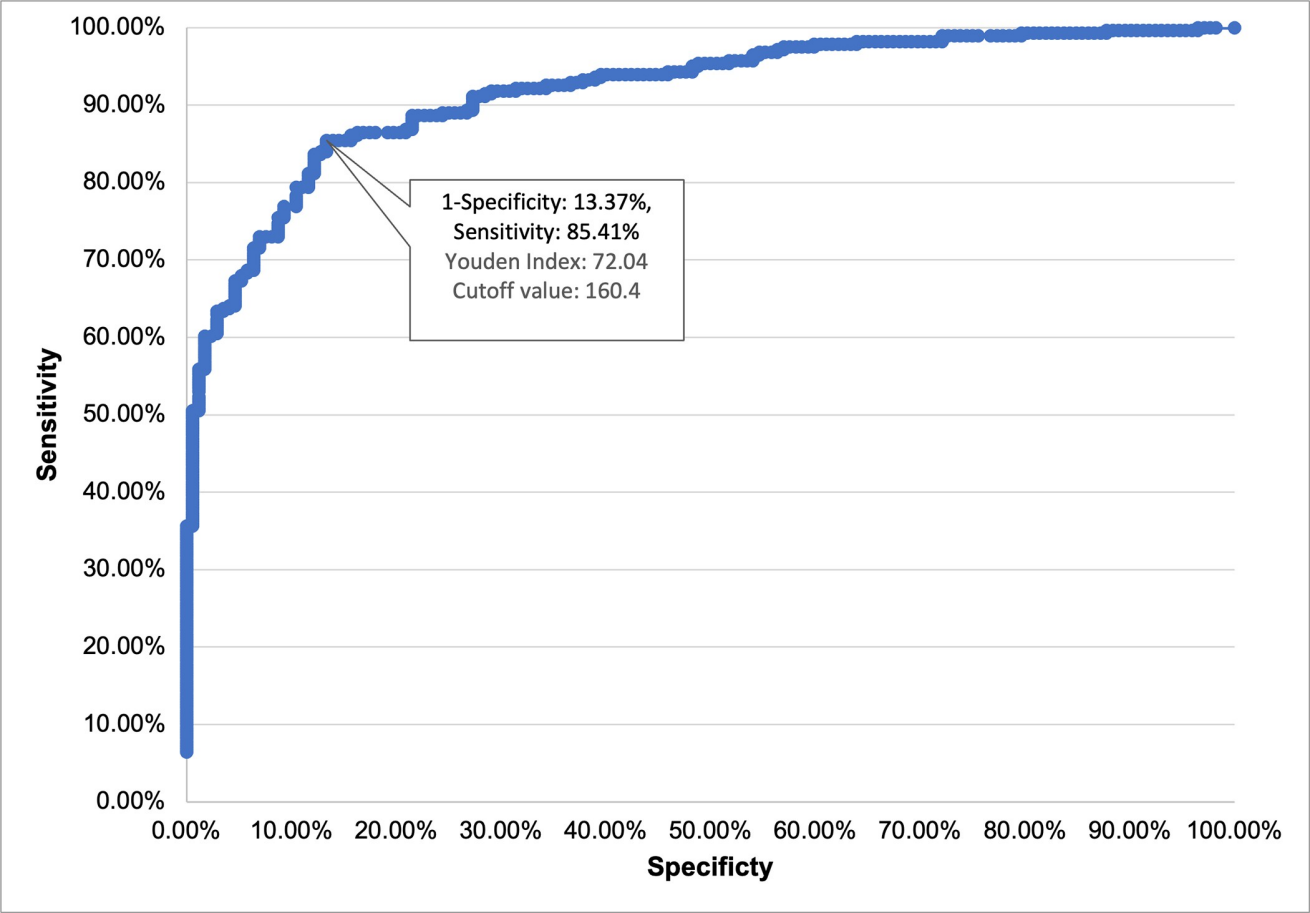
ROC analysis and Youden index of the cutoff value.

The cutoff value was also verified using ROC analysis and the Youden index (14,15). This yielded the same optimal cutoff point, as shown in Fig 4. It is important to note that this value is an indication of the cutoff value at which both the qualitative and quantitative tests give reactive results. Therefore, it does not determine the accuracy of the tests used and is not conclusive of a definite level of protection against infection.

A total of 453 participants participated in both qualitative and quantitative COVID-19 antibody testing. Table 3 compares the qualitative results against the quantitative results using the 160.4 U/ml threshold.

**Table 3:** Comparison of the qualitative and quantitative results of all vaccine brands (N = 453).

Quantitative Count (U/ml)	Positive	Negative
≥ 160.4	240	23
< 160.4	41	149

### Vaccine brand IgG reactivity

Since the first sample collection in June until December 2021, 313 (23.7%) (participants received their first dose, 885 (67.1%) were fully vaccinated, and 120 (9.1%) were unvaccinated. Most of the participants (48%) received CoronaVac as their vaccine followed by AstraZeneca at 15% of participants. Based on the qualitative test kit administered, it was observed that the Pfizer vaccine, an mRNA vaccine, produced the highest amount of antibodies, followed by Moderna (mRNA vaccine), AstraZeneca (viral vector vaccine) and CoronaVac (inactivated vaccine). Those who tested positive on the IgG test were 58.1x more likely to have received the Pfizer vaccine, and so on. Regardless of vaccine brand, vaccinated individuals had an increased odds of having reactive IgG serology values (Table 4).

**Table 4:** IgG reactivity with vaccine brand based on the qualitative antibody test (N = 1,198).

Vaccine Brand	N (%)	IgG Reactive Serology (%)	95% CI for IgG Reactive Serology		p	OR (95% CI)
			Lower Bound	Upper Bound		
AstraZeneca	198 (15)	139 (70.2)	0.633	0.765	<0.01	12.7 (7.1–22.7)
Moderna	59 (4.5)	53 (89.8)	0.792	0.962	<0.01	49.5 (16.0–153.0)
Pfizer Biontech	46 (3.5)	43 (93.5)	0.821	0.986	<0.01	58.1 (16.4–206.5)
CoronaVac	633 (48)	327 (51.7)	0.477	0.556	<0.01	5.0 (3.0–8.2)

### Predictors of IgG reactivity

Possible risk factors affecting IgG serology were examined. These included gender, age, region of residence, educational attainment, work arrangement, cigarette smoking status, and the presence of co-morbidities like diabetes, hypertension, obesity and asthma. Household conditions (i.e. if there were vulnerable individuals or frontliners in the household or vaccinated individuals) were also examined to see if they affected IgG serology. Participants with obesity or those who are smokers were found to be 1.7x and 1.8x more likely to have less reactive IgG serology results, respectively. Other risk factors were not significantly associated with IgG reactivity (Table 5).

**Table 5:** Adjusted odds ratios of risk factors affecting IgG serology among study participants.

Risk Factor	aOR* (95% CI)	p
Sex	1.3 (1.0–1.8)	0.07
Age	1.0 (0.9–1.0)	0.69
Region of Residence	1.1 (0.7–1.6)	0.71
Highest Educational Attainment	0.8 (0.5–1.2)	0.29
Work Arrangement	0.8 (0.6–1.1)	0.12
Comorbidities		
With at least 1 comorbidity	1.0 (0.7–1.6)	0.92
Diabetes	0.9 (0.5–1.4)	0.54
Hypertension	0.7 (0.4–1.1)	0.14
Obesity	0.6 (0.4–1.0)	0.05*
Asthma	0.8 (0.4–1.4)	0.41
Smokers	0.5 (0.3–0.9)	0.02*
Household Testing Positive	1.2 (0.5–2.8)	0.61
At Least 1 Household Individual Vaccinated	1.5 (0.9–2.3)	0.07
Vulnerable Individuals in Household	0.9 (0.7–1.2)	0.46
Frontliners In Household	1.3 (0.9–1.8)	0.10

*aOR—adjusted odds ratio.

## Discussion

Seroprevalence is an estimate of people who have developed antibodies against a pathogen. In a review by Post and colleagues, it was found that seroconversion of IgM antibodies occurred any time from 4 to 14 days after infection and declined 6 weeks after disease onset [20]. On the other hand, IgG antibodies were detected from 12–15 days after infection, and declined after 8 weeks.

This study revealed that the seroprevalence rate of COVID-19 antibodies ranged from 28.76% to 65.15% among members of a university, from June to December 2021. There was no significant difference in seroprevalence by gender and age in the study population. Seropositivity in the analysis was mainly due to the antibody response developed due to vaccination. These values are consistent with results published regarding COVID-19 vaccine development. IgG titers against the SARS-CoV-2 S protein, spike subunit, and receptor binding domain have previously been measured in individuals with mRNA vaccines, developed by Pfizer Biontech or Moderna [21–24]. It was found that more than 97% of individuals developed IgG titers after their first dose, and 100% of them developed antibodies after their second dose. In our study, mRNA vaccines had a seropositivity rate of 89.8%- 93.5% which is a little lesser than studies by the manufacturer. Studies conducted on the vaccines with the inactivated virus yielded a similar increase in seropositvity. A study on 1,247 healthcare workers in Brazil showed that there was an increase in seropositivity rates after the first dose (88%), and an even higher seropositivity rate after the second dose (99%) [23]. In the study, vaccination with CoronaVac, an inactivated vaccine, showed a 51.7% positivity rate, which is a value much lower than the values published in the Brazil study.

Although vaccination rates were high across the study population, a decreasing trend in the seroprevalence was found over time after vaccination. After the development of an antibody response to the S protein of the virus, a decline in antibody levels has likewise been observed after 3 months and after 6 months of the second dose in several studies [21, 25, 26]. This may explain the decline in seroprevalence found in the results over time. This decrease of antibodies over time supports the need for booster shots that was recommended by the DOH. This also supports the continuous use of health precautions like use of masks, frequent handwashing and social distancing.

The qualitative test was done on all participants of the study. This showed that Pfizer had the most number of reactive IgG results (Pfizer 93% vs Moderna 89.8%). The quantitative test was done on only 453 participants of the study. This still showed that Pfizer had higher seropositivity rates using the 160.4 U/ml cut-off. However, Moderna showed higher mean antibody titers compared to Pfizer (Moderna: 7916.8 vs. Pfizer: 3292.2). This shows that following the cut-off computed from this study, Pfizer had more seropositivity rates although Moderna had higher antibody production among those who seroconverted. The confidence intervals of the rate of IgG seropositive participants with the Moderna and Pfizer vaccines overlap, so the difference between the two groups are not statistically significant. The qualitative testing determines the presence or absence of IgG and IgM antibodies against the nucleocapsid protein of SARS-CoV-2 [16, 27]. The quantitative test targets antibodies against the spike protein, and it results in a numerical value of the antibody titers detected [27]. Furthermore, the difference in antigen content, the number of doses, and intervals between doses have previously been reported as potential reasons for the varying immune response between Moderna and Pfizer [28, 29].

Among the risk factors included in the study, only obesity and smoking status were significantly associated with IgG serology. Similar to this, a seroprevalence study in Switzerland [9] and Russia [30] showed that current smokers were less likely to have reactive serology results compared to non-smokers, though these studies only focused on seropositivity after infection. Chronic health conditions were also not significantly associated with serology results in the Switzerland population [9]. However, contrary to our results, this study also reported that obese women had higher odds of receiving reactive serology results compared to non-obese women, but there were no differences observed in male obese participants. A cross-sectional seroprevalence survey in Abu Dhabi also resulted in similar results, with smokers showing lower seroprevalence [31]. BMI categories and comorbidities did not show any significant associations with seropositivity. In another study, BMI was associated with antibody titer levels. Specifically, obese participants exhibited a reduced humoral immune response compared to under-weight and normal-weight individuals [32]. An article by Westheim and colleagues tackles potential causes of reduced immunogenicity post-vaccination in obese patients [33]. High levels of leptin in obese individuals lead to the downregulation of activation-in induced cytidine deaminase (AID) and E47 in B cells [34]. Obesity was correlated with reduced humoral memory B cell response, which effectively reduces the duration of the protection of vaccines against COVID-19 infection [35]. In a separate study involving obese individuals infected by SARS-CoV-2, it was concluded that IgG serology was negatively associated with pulmonary inflammatory markers, specifically serum amyloid A protein (SAA), C-reactive protein (CRP), and ferritin [35, 36]. These markers induce pro-inflammatory events that cause the overproduction of inflammation markers, and it leads to a disturbed B cell response due to local and systemic inflammation. Emerging evidence suggests that vaccine-induced antibody titers in smokers decrease at a faster rate compared to non-smokers [37]. A reduced antibody response to COVID-19 vaccines have also been found in smokers, regardless of the overall duration or amount of smoking per day [38]. It is hypothesized that smoking impairs the ability of the immune system to produce memory cells [37, 39]. Previously, smoking has been linked with an increase in monocyte macrophage counts which potentially reduce the amount of circulating antibodies [37, 40]. Some studies attributed this decrease in antibody production and quicker lowering of IgG titers to the presence of nicotine. Nicotine is hypothesized to prevent antibody production and impair T cell signaling [41]. However, the mechanism of how smoking affects antibody production and overall immune system response is yet to be elucidated [37].

This study was limited to the measurement of antibodies against SARS-CoV-2 in a university population. It was important to note that antibody seroprevalence varied depending on the location, target participants, and timing in which the testing was conducted during the pandemic [42]. The seroprevalence obtained in this study was only reflective of the status of the university community at the time of testing. Antibody testing was not used to diagnose infection in individuals, nor the amount of protection offered against COVID-19 infection. At the moment, there was no consensus on the best approach used to evaluate COVID-19 infection status in relation to a university community [43, 44]. Donneau and colleagues recommended the use of a saliva RT-PCR test and antibody test in conjunction with self-reporting of symptoms in order to determine the prevalence of COVID-19 in the population and characterize seroconversion and seroreversion after vaccination and infection [45]. A case study on Cornell University used 3 mechanisms to limit transmission: testing, contact tracing, and symptomatic self-reporting. The study recommended that universities test students at least once per week, regardless of vaccination rates and status [46]. Similar recommendations were provided in modeling studies conducted at Duke and Harvard Universities. In addition, other preventive measures, such as campus-based screening, quarantine protocols, hygienic measures, and cafeteria and dormitory regulation, were also necessary for the safe reopening of universities [47]. Local universities may continue to monitor COVID-19 antibody status as a measure of vaccination status for an epidemiological situational analysis. Antibody testing was reserved for research purposes only.

## Conclusions

This study conducted among administrators, faculty, staff and students in a private tertiary university in the Philippines showed a SARS-CoV-2 antibody seroprevalence range of 28.8% to 65.1%. We observed a decreasing trend of antibody levels over a six-month observation period after vaccination. IgG antibody formation was observed in all brands of vaccines. Among the brands, antibody reactivity was highest in Pfizer, followed by Moderna, AstraZeneca and CoronaVac. It is recommended for future research to focus on the distribution of quantitative anti-SARS-CoV-2 IgG responses for both pre- and post- vaccine exposures for the different vaccine brands. We also recommend future endeavors to identify if antibody titer levels has any significant association with individuals who have chronic pre-existing medical conditions.

This study suggests that where possible, IgG and T-cell reactivity and/or neutralizing capacity against SARS-CoV-2 need to be monitored regardless of vaccine brand. Together with uptake of COVID-19 vaccines and boosters, other public health interventions including wearing of masks, frequent hand washing, social distancing and regular testing should be continued. Effective communication is also needed to inform risks associated with activities across different settings. Investments in long-term measures such as improving air filtration and ventilation systems need to be made. Institutions in the Philippines may consider establishing responsive surveillance systems including wastewater surveillance for earlier detection and rapid response to spikes in COVID-19 in the community.
